# Clinical, epidemiological, and care profile of hospitalized patients with retinoblastoma in Brazil

**DOI:** 10.5935/0004-2749.2023-0073

**Published:** 2024-07-09

**Authors:** Annamaria Ciminelli Barbosa, Maria Clara de Magalhães-Barbosa, Giovanni Nicola Umberto Italiano Colombini, Arnaldo Prata-Barbosa

**Affiliations:** 1 Department of Pediatrics, Instituto D’Or de Pesquisa e Ensino, Rio de Janeiro, RJ, Brazil; 2 Department of Ophthalmology, Universidade Federal do Estado do Rio de Janeiro, Rio de Janeiro, RJ, Brazil; 3 Instituto de Puericultura e Pediatria Martagão Gesteira, Universidade Federal do Rio de Janeiro, Rio de Janeiro, RJ, Brazil

**Keywords:** Retinoblastoma/diagnosis, Retinoblastoma/epidemiology, Patient care, Humans, Children, Adolescents, Brazil

## Abstract

**Purpose:**

To describe the epidemiological and clinical profile of hospitalized patients with retinoblastoma in Brazil.

**Methods:**

Using data from the Hospital Cancer Registry of the *Instituto Nacional de Câncer,* patients with the morphological codes of retinoblastoma who were diagnosed between 2000 to 2018, aged 0–19 years, and followed up in registered hospitals (analytical cases) were selected. The relative and absolute frequencies of demographic, clinical, diagnostic, therapeutic, and outcome variables were described. Hospital performance indicators were calculated and compared between hospitals qualified and not qualified to treat pediatric oncology cases and between hospitals with different case volumes (<20, 20-75, >75 cases).

**Results:**

Of the 2,269 identified analytical cases from 86 institutions, 48% were from the Southeast, 54% were male, and 66% were aged <4 years. The proportion of missing data (NA) was too high for several variables. Approximately 84% of the patients were from the public health system, 40% had a positive family history, and 88% had unilateral involvement. The first treatment included surgery in 58.3% of the patients (NA=2), Approximately 36.6% of these patients achieved complete remission, 10.8% achieved partial remission, and 12.7% died (NA=59%). Hospital performance indicators were within the target in >90% of the patients. The median time between the first appointment and diagnosis (6 days, interquartile range [IQR] 1–14) was significantly lower and the median time to death was longer (343 days, IQR, 212-539) in high-volume hospitals (>75 cases) than in medium- and low-volume hospitals.

**Conclusions:**

Despite the high proportion of missing data, we found that the delay in diagnosis is due to prehospital factors. Additionally, there is a need for educational programs for healthcare professionals and families that emphasize early identification and referral to specialized centers. Future studies should focus on the impact of Hospital Cancer Registry data completeness on outcomes, causes of delay in diagnosis, regional inequalities, and barriers to accessing specialized services.

## INTRODUCTION

Retinoblastoma is the most common malignant ocular tumor seen in children. It arises due to the inactivation of both alleles of the *RB1* tumor suppressor gene (chromosome 13q), resulting in the formation of a defective protein called pRB, which causes cell cycle alterations and disordered cell proliferation^(1)^. The most frequently observed clinical findings are leukocoria, strabismus, and low vision, which may have a unilateral or bilateral presentation. Inflammatory signs such as hypopyon, ocular hyperemia, and even proptosis may appear^(2)^. It mainly affects children aged 0–4 years, with no preference for sex or race. In this age group, in high-income countries, the incidence ranges from 10 to 12.1/1,000,000 children or approximately 1/17,000 live births^(3,4,5,6)^. Data are poor or less consistent in low- and middle-income countries, with reportedly lower incidence rates, such as 4.7 in Mexico^(7)^, 5.3 in Pakistan^(8)^, 7.13 in Brazil^(9)^, 7.62 in Lebanon^(10)^, and 7.7 in South Africa^(11)^, per 1,000,000 children in the same age group.

Improvements in diagnostic and therapeutic methods have resulted in higher patient survival rates, especially in high-income countries (almost 99%). There are numerous therapeutic options today, such as intraarterial chemotherapy, brachytherapy, and laser photocoagulation. Together, these therapies can preserve the eyeball and life^(21)^. However, retinoblastoma can still be fatal and associated with a poor prognosis if it is diagnosed late and not treated adequately. Low- and middle-income countries still have high rates of eyeball enucleation and mortality, which are primarily related to late diagnosis of retinoblastoma in advanced and metastatic stages^(31)^.

Worldwide, numerous health policies aim to monitor patients with retinoblastoma. In Brazil, the main database is the *Registro Hospitalar de Cancer* (RHC) of the *Instituto Nacional de Câncer* (INCA). Epidemiological studies in the Brazilian population have mainly been conducted in individual institutions; a few have been conducted to assess the quality of care in the country^(13,14)^. In this study, we aimed to describe the epidemiological, clinical profile, and hospital quality of care indicators of patients diagnosed with retinoblastoma between 2000 and 2018 who were included in Brazil’s RHC. We hope that the study findings will contribute to elucidating the care profile for patients with retinoblastoma, identifying difficulties in the line of care, and understanding the impact of the disease in Brazil. This may help further develop effective public health policies to improve patient care.

## METHODS

### Design, population, and period of study

This was a descriptive retrospective study of hospitalized patients aged 0-19 years who were diagnosed with retinoblastoma from 2000 to 2018 in Brazil and were included in the RHC/INCA database.

### Characteristics of the RHC

The RHC/INCA is a national comprehensive database that collects information regarding the diagnosis, treatment, epidemiological profile, and evolution of malignant neoplasms. Hospitals qualified for cancer care by the Brazilian *Sistema Único de Saúde* (SUS) are required to send data to the RHC. According to the 2010 RHC Manual, the percentage of unregistered patients is insignificant and corresponds to those being treated at private health centers, from which data sharing is optional. The system uses the International Classification of Diseases (ICD) - Tenth Revision (lCD-10), ICD for Oncology - second and third edition (1CD-O2 and 1CD-O3), Classification of Malignant Tumors - sixth edition (TNM), International Classification of Childhood Cancer, Classification for Tumors in Adolescents and Young Adults (CAAJ), and the identification codes of Brazilian municipalities from the *Instituto Brasileiro de Geografia e Estatística.*

### Data collection

Data were obtained on July 2021 (https://www.inca.gov.br/numeros-de-cancer/registros-hospitalares-de-cancer-rhc), using the following filters: age, 0-19 years; 1CD-10 code, C69.2 for retinoblastoma; first diagnosis, 2000-2018; and 1CD-O2 and 1CD-O3 codes for histological type, 9510/3 (retinoblastoma, NOS - not otherwise specified), 951 1/03 (differentiated retinoblastoma), 9512/3 (undifferentiated retinoblastoma), and 9513/3 (diffuse retinoblastoma).

### Data analysis

For demographic, clinical profile, and care features, only the following analytical cases (as described in the RHC Manual) were included: a) patients diagnosed (or not) in the hospital who completed their treatment and were followed up at the RHC hospital; b) patients diagnosed at the RHC hospital whose treatment was initiated at another hospital (recommended as per the plan of the RHC hospital doctors), but who returned to the RHC hospital for treatment completion and follow-up; and c) patients diagnosed at another hospital where specific antineoplastic treatment was started, but who completed their treatment and were followed up at the RHC hospitale.

Performance indicators, such as the process, productivity and quality indicators, were evaluated. The process indicators (volume of care in RHC hospitals and information quality) included the following: number of new cases registered in the period; percentage of analytical cases; percentage of patients who started the diagnosis and treatment process at the hospital; percentage of patients who arrived at the hospital with advanced disease and without a diagnosis; and percentage of patients without information on certain variables such as clinical condition at the beginning of treatment, previous diagnosis and treatment, most important diagnostic bases, staging, main reason for not undergoing the first treatment at the hospital, first treatment received, and disease status at the end of the first treatment. The productivity indicators, which are related to the hospital’s efficiency in treating patients, were estimated in patients who started the diagnosis and treatment process at the RHC hospital. They included the median time between (i) screening and the first appointment at the clinic that administered the treatment, (ii) first appointment and the first confirmed diagnosis, (iii) first confirmed diagnosis and the beginning of the first antineoplastic treatment, (iv) screening and the initiation of the first anticancer treatment, and (v) first confirmed diagnosis and death. The quality indicators, which are related to the effectiveness of care at the RHC hospital, included the following: percentage of analytical cases without evidence of the disease at the end of the first antineoplastic treatment and death rate in the first year after diagnosis.

The median time intervals between first appointment and the first confirmed diagnosis, first confirmed diagnosis and the beginning of the first antineoplastic treatment, and (v) first confirmed diagnosis and death were estimated in hospitals qualified and not qualified for treating pediatric oncology cases and compared using the Mann-Whitney test. These intervals were also estimated and compared using the Kruskal-Wallis test, according to the volume of cases treated in each hospital (<20, 20 to 75, and >75). The significance level was set at 0.05. All analyses were performed using GraphPad (version 9.5.1; Dotmatics, Boston, MA, USA).

## RESULTS

From 2000 to 2018, 2,821 patients with retinoblastoma from 104 hospitals were registered in the RHC. Of these, 2,269 (80%) patients from 86 hospitals were classified as analytical cases, which included 1,084 (47.8%) patients from 37 hospitals (43.0%) in the Southeast, 604 (26.7%) patients from 20 hospitals (23.3%) in the Northeast, 252 (11.2%) patients from 16 hospitals (18.6%) in the South, 182 (8.0%) patients from eight hospitals (9.3%) in the North, and 142 (6.3%) patients from five hospitals (5.8%) in the Midwest.

Several demographic, clinical, diagnostic, and therapeutic variables had significant proportions of missing data. According to the available data, 54% of the patients were male, 66% were aged 1–4 years, 50% were brown, 84% were from SUS-approved hospitals, 40% had a positive family history, 54% had not been previously diagnosed or undergone treatment, 88% had unilateral ocular involvement, and 91% had only one tumor at the time of diagnosis (Tables 1 2). The staging data were too inconsistent to estimate its distribution.

**Table 1:** Demographic characteristics of the analytical cases of retinoblastoma

Characteristics	n	%^a^	95% CI
Sex			
Male	1,234	54.4	52.3 – 56.4
Female	1,035	45.6	43.6 – 47.7
Age range			
<1 year	614	27.1	25.3 – 28.9
1–4 years	1,502	66.2	64.2 – 68.1
5–9 years	138	6.1	5.2 – 7.1
10–14 years	12	0.5	0.3 – 0.9
15–19 years	3	0.1	0.04 – 0.4
Race			
Brown	570	50.3	47.4 – 53.2
White	488	43.0	40.2 – 45.9
Black	61	5.4	4.2 – 6.8
Asiatic	12	1.1	0.6 – 1.8
Indigenous	3	0.3	0.1 – 0.8
No information	1,135	-	
Patient education level			
None	1,813	94.6	93.5–95.5
Not completed elementary school	90	4.7	3.8–5.7
Completed elementary school	7	0.4	0.2–0.8
Middle school	4	0.2	0.1–0.5
Not completed higher education	1	0.05	0.0–0.3
Completed higher education	1^b^	0.05	0.0–0.3
No information	353	-	
Region of residence			
North	182	8.0	7.0–9.2
Northeast	604	26.7	24.9 – 28.5
Southeast	1084	47.8	45.8–49.9
South	253	11.2	9.9–12.5
Midwest	142	6.3	5.3–7.3
No information	4	-	-
Patient referred by			
Public unified health system	926	84.3	82.1–86.4
Private or philanthropic health system	154	14.0	12.1–16.2
Own account	18	1.7	1.0–2.6
No information	1,171	-	
TOTAL	2,269	100.0	

a Frequencies were calculated based on reported data (excluding missing values)

b Probably incorrect registry

CI, confidence interval

**Table 2:** Clinical characteristics of the analytical cases of retinoblastoma

Features	n	%^a^	95% Cl
Family history			
Yes	252	39.8	36.1–43.7
No	381	60.2	56.3–63.9
No information	1,636		
Previous situation			
No diagnosis and no treatment	1,194	53.9	51.8–55.9
With diagnosis and without treatment	921	41.6	39.5–43.6
With diagnosis and treatment	71	3.2	2.5–4.0
Others	30	1.4	0.9–1.9
No information	53	-	
Laterality			
Right	721	44.9	42.5–47.4
Left	698	43.5	41.1–45.9
Bilateral	186	11.6	10.1–13.2
No information	664	-	
Presence of more than one tumor			
No	1,112	90.8	89.0–92.3
Yes	110	9.0	7.5–10.7
Doubtful	3	0.2	0.1–0.7
No information	1,044	-	
TOTAL	2,269	100.0	

a Frequencies calculated based on the total number of reported features (excluding missing values).

CI, confidence interval.

Pathological examination was the leading test for diagnosing retinoblastoma (70.7%), and the primary tumor histology was the most important basis for the diagnosis (68%). However, both variables had >50% missing data. The first treatment (only two missing values) was chemotherapy (74.5%) or surgery (58.3%, no reported type), and they were usually associated with other therapies. At the end of the first treatment at the hospital, 36.6% of the patients achieved complete remission, 10.8% achieved partial remission, and 12.7% died during the study period (59% of the values were missing). Deaths were computed when the “date of death” cell was filled in (303 patients, 13.4%). Empty cells were considered undead (Table 3).

**Table 3:** Diagnostic and therapeutic characteristics of the analytical cases of retinoblastoma

Studied variable	n	%^a^	95% CI
Tests relevant to the diagnosis			
Clinical examination and clinical pathology	17	1.7	1.0–2.7
Imaging exam	242	23.8	21.3–26.6
Exploratory surgery	10	1.0	0.5–1.8
Pathologic anatomy	718	70.7	67.9–73.4
Tumor markers	28	2.8	1.9–4.0
No information	1,254	-	1.9–3.8
Most important basis for diagnosis			1.5–3.1
Clinical	33	2.7	23.9–28.8
Clinical research	26	2.1	0.0–0.5
Imaging exam	320	26.3	0.4–1.4
Tumor markers	1	0.1	0.0–0.5
Cytology	9	0.7	65.3–70.5
Metastasis histology	1	0.1	
Primary tumor histology	828	68.0	
No information	1,051	-	
First treatment at the hospital (Isolated or combined)			
Chemotherapy	1,689	74.5	72.7–76.3
Surgery	1,322	58.3	56.3–60.3
Radiotherapy	351	15.5	14.1–17.0
Other	266	11.7	10.5–13.1
None	43	1.9	1.4–2.5
No information	2	-	
Disease status at the end of the first hospital treatment			
Complete remission	343	36.6	33.6–39.7
Partial remission	101	10.8	9.0–12.9
Stable disease	225	24.0	21.4–26.9
Disease in progress	87	9.3	7.6–11.3
Oncological therapeutic support	12	1.3	0.7–2.2
Death	119	12.7	10.5–15.0
Not applicable	50	5.3	4.1-7.0
No information	1,332	-	
Date of death			
With date	303	13.4	12.0–14.8
No information	1,966	86.6	85.2–88.0
TOTAL	2,269	100.0	

a Frequencies calculated based on the total number of reported features (excluding missing values).

CI, confidence interval.

**Figure 1:**
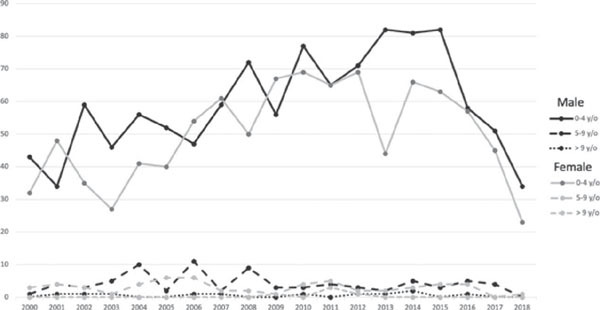
Annual distribution of analytical cases of retinoblastoma according to sex and age that were diagnosed between 2000 to 2018 according to data from Brazil’s Hospital Cancer Registry

Until 2014, there was an increase in the number of patients screened (37%), diagnosed (33%), who attended their first appointment (33%), in whom specific treatment was initiated (33%), and who died (28.4%) (Table 4). The annual distribution of the analytical cases according to sex and age are shown in figure 1. Approximately 54% of the analytical cases began the diagnosis and treatment process at a registered hospital. We could not estimate the proportion of patients who arrived with an advanced disease or without a diagnosis. The median time between screening and the first appointment, first appointment and the confirmed diagnosis, confirmed diagnosis and the beginning of the first antineoplastic treatment, and screening and the initiation of the first anticancer treatment were within the RHC goals in >90% of the reported cases (50% of screening data were missing). The mortality rate of the 1,194 cases that presented without a diagnosis or having undergone any treatment was 13.2%; approximately, 61.4% of these deaths occurred in the first year of diagnosis (Table 5).

**Table 4:** Distribution of care for the analytical cases of retinoblastoma

Variable	Retinoblastoma cases		Registered institutions
	N	%^a^	n
Year of first diagnosis			
2000–2004	463	20.7	38
2005–2009	605	27.0	60
2010–2014	731	32.7	60
2015–2018	436	19.5	49
No information	34	-	11
Total number of reported cases	2,235	100.0	
Year of screening			
2000–2004	217	19.4	24
2005–2009	309	27.6	36
2010–2014	416	37.1	39
2015–2018	178	15.8	34
No information	1,149	-	35
Total number of reported cases	1,120	100.0	
Year of first appointment			
2000–2004	465	20.5	37
2005–2009	611	26.9	59
2010–2014	748	33.0	58
2015–2018	445	19.6	50
Total number of reported cases	2,269	100	
Year of initiation of the first specific treatment for the tumor			
2000–2004	451	20.3	36
2005–2009	599	27.0	58
2010–2014	723	32.7	53
2015–2018	442	19.9	49
2019	2	0.1	2
No information	52	-	24
Total number of reported cases	2,217	100.0	
Year of death			
2000–2004	65	21.5	16
2005–2009	78	25.7	25
2010–2014	86	28.4	30
2015–2018	65	21.5	25
2019	8	2.6	5
2020	1	0.3	1
No information	1,966	-	
Total number of deaths	303	13.4	

a Frequencies calculated based on the total number of reported cases (excluding missing values).

**Table 5:** Registro Hospitalar de Cancer (RHC) performance indicators associated with retinoblastoma diagnosis during the study period (2000–2018)

Indicators Process indicators	Formula	Results	
		n/Total	%
Patients that started the diagnosis and treatment process in the hospital (%)	Patients w/o diagnosis and treatment (n) × 100 total cases	1,194/2,821 1,194/2,269	42.9% 53.9%
**Productivity indicators** ^a^	**Goals recommended by the RHC**	**n (%)**	**Median (days) OQR 25–75)**
Median time interval between the screening and the first appointment (n=633)	<30 days ≥30 days NI	618 (98%) 15 (2%) 561	0 (0–0) 116 (63–267)
Median time interval between the first appointment and the first confirmed diagnosis (n=1177)	<60 days ≥60 days NI	1,093 (93%) 84 (7%) 17	7 (1–16) 105.5 (72–182)
Median time interval between date of first diagnosis and first antineoplastic treatment (n = 1057)	<30 days ≥30 days NI	963 (91%) 94 (9%) 137	3 (0–10) 48.5 (34–87)
Median time interval between screening and first antineoplastic treatment (n=632)	<120 days ≥120 days NI	595 (94%) 37 (6%) 561	17 (9–32) 189 (156–284)
**Quality indicators** ^a^	**Goals recommended by the RHC**	**n/N**	**%**
Deaths in the first year (%) **§** (among 2269 analytical cases)	<365 days >365 days Total	183/298 115/298 298/2269	61.4% 38.6% 13.1%
% Deaths in the first year **§** (Among 1194 analytical cases arriving w/o diagnosis and treatment)	<365 days	>365 days	Total
102/158	56/158	158/1194
64.6%	35.4%	13.2%

RHC, Hospital Cancer Registry; IQR, interquartile range.

a Calculated only for analytical cases.

‡ Of the 1194 analytical cases that arrived at the hospital without a diagnosis and treatment, 69 had no information regarding the Reese-Ellsworth staging.

§ The number of deaths must be interpreted with caution, as it was extracted from the “date of death” field. 1n cases where this variable was not completed, it was not possible to guarantee that no death had occurred.

**Figure 2:**
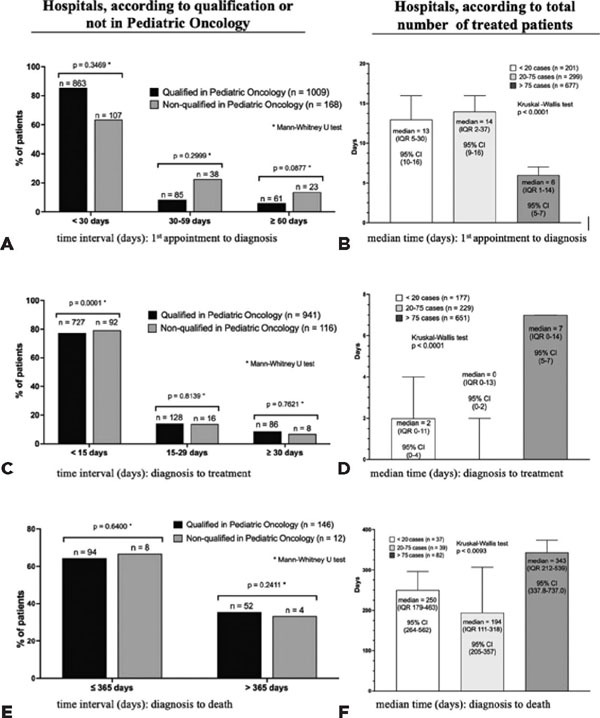
Percentage of patients with retinoblastoma for time-dependent variables according to the type of hospitals (qualified or not for treating pediatric oncology cases) and volume of cases treated. Data of hospitals qualified or not for treating pediatric oncology cases were compared using the Mann-Whitney U test (left side). Data of hospitals with different volume load were compared using the Kruskal-Wallis test (right side).

The percentage of cases treated in hospitals qualified for pediatric oncology (QPO) varied among regions: South, 97.5%; Southeast, 95.6%; North, 89.9%; Northeast, 72.3%; and Midwest, 48.2%. Although the percentage of patients with <30 days between the first consultation and diagnosis was higher in QPO hospitals than in non-QPO hospitals, the difference was not significant (p=0.3469, Figure 2A). However, hospitals with high volume of care (>75 cases) demonstrated a significantly shorter median time interval (6 days, interquartile range [1QR] 1-14) than those with medium and low volume of care (p<0.001, Figure 2B). QPO hospitals had a significantly lower percentage of patients (p=0.0001, Figure 2C) than non-QPO hospitals in whom treatment was initiated <15 days after diagnosis. This median time interval was significantly longer (7 days, 1QR 0–14) in high-volume hospitals than medium- and low-volume hospitals (p<0.0001, Figure 2D). However, 75% of patients in all the hospitals had an interval of <15 days. No difference was observed between QPO hospitals or in the proportion of patients who died (Figure 2E). However, median survival was longer in high-volume hospitals (343 days, 1QR 212–539) than in in medium- and low-volume hospitals (p<0.0093, Figure 2F).

## DISCUSSION

The present study, which used data from the RHC/1NCA in Brazil, demonstrated that most cases of retinoblastoma occurred in the age group of 1-4 years, with a slight predominance in males. We also found high proportions of missing values in several variables. Most reported cases had unilateral involvement and no significant family history. Pathological anatomy was primarily used to confirm the diagnosis. 1n most patients, treatment included chemotherapy and surgery. The mortality rate was high, and most deaths occurred in the first year of diagnosis. Time-dependent indicators were within the target established in >90% of the patients, which suggests that the quality of in-hospital care is good.

The probable cause of missing data was failure to complete the RHC or medical record. This suggests that the data may be missing at random, resulting in similar distributions of variables for the reported and missing data. However, it is essential to host regular training programs to raise awareness about the importance of correctly filling out data to generate accurate information.

The demographic and clinical features of this study’s population are in accordance with those of several worldwide studies^(6,15)^. Most patients were from the Southeast region, which is the wealthiest, most populated, and most developed region, with the most significant number of oncology hospitals. However, it is also possible that this region received many referred patients from less developed areas. However, previous studies have reported the highest incidence of retinoblastoma from the Northeast region (e.g., Natal and Salvador), which is socioeconomically less favored^(9)^. Some studies have identified an association between retinoblastoma prevalence and unfavorable environmental risk factors, such as coinfection with HPV^(16)^, maternal diet, and low folate intake^(17)^.

In this study, inconsistent data on tumor staging prevented its reliable estimation. Other studies have demonstrated high percentages of advanced-stage diagnosis in the Brazilian population^(18,19,20,21)^. Chantada et al.^(22)^ demonstrated that patients with familial retinoblastoma from developing countries (Argentina, Brazil, Jordan, Turkey, and Venezuela) were diagnosed significantly later and with a more advanced intraocular disease and an increased risk for bilateral enucleation than patients from the USA (only 25% of patients presented with stage 5 retinoblastoma). This reflects a delay in diagnosis, which may be multifactorial. Mattosinho et al.^(13)^ demonstrated that maternal education and time between the first consultation and referral were significantly related to advanced-stage diagnosis and survival in patients with retinoblastoma presenting to INCA, Rio de Janeiro. The time between the first symptoms and referral to an oncology center accounts for an alarming percentage (70%) of the overall gap between diagnosis and treatment in Brazil, compared with 23% in other developing countries^(23)^. These findings suggest that primary care physicians in the Brazilian health system need to be made more aware of retinoblastoma. Therefore, health programs should emphasize awareness among family members and medical professionals of the signs and symptoms of retinoblastoma to allow for early diagnosis and a better prognosis.

Pathological anatomy and primary tumor histology were the main diagnostic methods in this study. Currently, retinoblastoma is clinically diagnosed using fundoscopy. Ocular ultrasonography and nuclear magnetic resonance imaging can be requested for tumor staging^(24)^. Histological examination of the tumor is reserved for patients in whom enucleation is necessary, such as those with a more severe disease and with a worse prognosis. Although our findings might suggest a delay in diagnosis, this conclusion could not be made because data were missing in approximately half of the patients. However, data regarding the first hospital treatment had only two missing values. Although there were no data on the type of surgery performed (enucleation, exenteration, or others), the percentage of surgeries performed (58.3%) was lower than the percentage of pathological anatomy (70.7%) or primary tumor histology (68.0%) data, which are diagnostic procedures that require a surgical specimen. This suggests that these pathologic diagnostic variables were overestimated. The percentage of surgeries performed in the present study is approximately the 3-year enucleation rate in high-income countries, according to a recent report (149 countries). The report revealed that the 3-year enucleation rates are much higher in low-income countries (reaching 73.6%) than in high-income countries (59.7%)^(25)^. This may reflect the lack of access to the most modern therapeutic technologies and the diagnosis of advanced-stage diseases in low-income countries^(25,26)^. With the availability of numerous less aggressive treatment options such as intraarterial chemotherapy, thermotherapy, and laser therapy, enucleation surgery is being performed less frequently and is reserved only for severe cases^(27,28)^.

The death rate in this study should be interpreted with caution. When the “date of death” was missing, it was impossible to rule out death. However, the overall case fatality rate was 13.4% among the analytical cases of retinoblastoma, which is high compared with that in high-income countries (0.8-1.0%)^(25)^. In the USA, the overall 5-year survival rate has demonstrated an upward trend, rising from 90.8% in the 1980s to 92.5% in the 1990s and 97.6% in the 2000s^(4)^. The case fatality rate reported in a few studies in Brazil has demonstrated a downward trend. It was 70% from 1956 to 1973 in Rio de Janeiro^(29)^, around 26% from 1975 to 1997 in Recife^(30)^, and 13% from 2006 to 2013 in Rio de Janeiro^(13)^. Among the analytical cases that arrived at the referral center without a diagnosis or having received any treatment, we found a case fatality rate of 13.2%. More than 60% of the deaths occurred in the first year, suggesting advanced-stage disease at diagnosis.

In this study, we observed an increase in the number of hospitalized cases of retinoblastoma until 2014, followed by a decrease in the number. This finding suggests a delay in updating the data in the RHC, which presents an opportunity for improvement. Continuous training in updating the RHC is essential to minimize failures in data collection and obtain a more reliable epidemiological profile of retinoblastoma.

The time-dependent indicators were quite positive, suggesting the good quality of in-hospital care. Most cases of retinoblastoma were treated in QPO hospitals, especially in the South and Southeast regions. In addition, although not significantly different, the percentage of patients with an optimal time interval between the first appointment and diagnosis was higher in QPO hospitals than in non-QPO hospital. Furthermore, this interval was significantly shorter in high-volume hospitals (>75 cases) than in medium- and low-volume hospitals. These findings suggest that the main component of the possible delay in diagnosis is the delay in referral to the tertiary center. The time interval between diagnosis and treatment was within the clinically acceptable range of 15 days in approximately 75% of the patients in all the hospitals. Public health campaigns should focus on raising awareness among family members and primary care professionals regarding the early signs of retinoblastoma and the need for prompt referral of patients to tertiary care centers to improve prognosis.

This study has some limitations inherent to the use of secondary data. Important variables had inconsistent information or no information in a large volume of cases, which could have introduced participation bias in addition to information bias. However, considering that the probable cause of missing data was failure to fill the RHC or medical records, there may be no difference in the characteristics of patients with missing data and those with complete data. However, this could not be demonstrated. Other variables associated with the stage at the time of diagnosis, such as maternal education, were not available. The date of first symptoms was also lacking and hampered the assessment of prediagnostic intervals, another variable associated with prognosis. In addition, the case fatality rate needs to be interpreted with caution because information regarding death depended on the “date of death” data. No casualty was considered when the field was empty, which could have resulted in an underestimation of the fatality rate.

Despite the high proportions of unreported data on retinoblastoma in the Brazilian RHC, the findings of the present study, such as the high mortality rates and positive hospital performance indicators, suggest that patients are presenting to specialized centers with advanced diseases and that the delay in diagnosis is related to prehospital factors. The study results highlight opportunities for the improvement on several fronts, such as incentives and ongoing training programs to fill out and regularly update RHC data, educational programs for primary care physicians and public health campaigns for families regarding the early signs of retinoblastoma, and policies to facilitate access to specialized centers. Future studies evaluating the impact of improving the completeness of RHC data on health outcomes, determining the causes of late diagnosis, assessing regional differences in retinoblastoma treatment, and determining the barriers to accessing specialized services could contribute to the development of strategies to modify the current situation and improve the disease prognosis in Brazil.
